# Accuracy and efficiency of drilling trajectories with augmented reality versus conventional navigation randomized crossover trial

**DOI:** 10.1038/s41746-024-01314-2

**Published:** 2024-11-10

**Authors:** Yao Li, Sergey Drobinsky, Paulina Becker, Kunpeng Xie, Myriam Lipprandt, Christian Andreas Mueller, Jan Egger, Frank Hölzle, Rainer Röhrig, Klaus Radermacher, Matías de la Fuente, Behrus Puladi

**Affiliations:** 1 Department of Oral and Maxillofacial Surgery, University Hospital RWTH Aachen, Aachen, Germany; 2 Institute of Medical Informatics, University Hospital RWTH Aachen, Aachen, Germany; 3 Chair of Medical Engineering, RWTH Aachen University, Aachen, Germany; 4 Department of Neurosurgery, University Hospital RWTH Aachen, Aachen, Germany; 5 Institute for Artificial Intelligence in Medicine, University Hospital Essen (AöR), Essen, Germany; 6 Center for Virtual and Extended Reality in Medicine (ZvRM), University Hospital Essen, University Medicine Essen, Essen, Germany

**Keywords:** Bone imaging, Outcomes research

## Abstract

Conventional navigation systems (CNS) in surgery require strong spatial cognitive abilities and hand-eye coordination. Augmented Reality Navigation Systems (ARNS) provide 3D guidance and may overcome these challenges, but their accuracy and efficiency compared to CNS have not been systematically evaluated. In this randomized crossover study with 36 participants from different professional backgrounds (surgeons, students, engineers), drilling accuracy, time and perceived workload were evaluated using ARNS and CNS. For the first time, this study provides compelling evidence that ARNS and CNS have comparable accuracy in translational error. Differences in angle and depth error with ARNS were likely due to limited stereoscopic vision, hardware limitations, and design. Despite this, ARNS was preferred by most participants, including surgeons with prior navigation experience, and demonstrated a significantly better overall user experience. Depending on accuracy requirements, ARNS could serve as a viable alternative to CNS for guided drilling, with potential for future optimization.

## Introduction

Drilling into bone along a trajectory is one of the most common yet most challenging tasks in medical interventions. However, it is not without risk, as important structures can be irreparably injured unintentionally during drilling. Its applications span multiple regions of the body, from the cranium to the jaw, shoulder, spine, pelvis, and ultimately to the extremities^1–9,10^. Although these procedures are all conducted on bone tissue and adhere to universal biomechanical principles^11,12^, the surgeons still often face significant challenges in maintaining drilling accuracy^13^, due to challenges of limited visibility of the underlying anatomy and plunging especially in minimal invasive surgery^14^. Limited visibility could obscure the view of critical anatomical landmarks, making it difficult for the surgeon to assign the entry point and difficult to navigate the instrument during drilling^15^. Plunging is defined as the penetration of the drill bit beyond the far cortex^8^, even experienced surgeons plunged over the far cortex by an average of 6.33 mm, thus causing inadvertent and iatrogenic damage to adjacent tissue^16^. The increasing improvement and spread of a variety of imaging modalities had paved the way for the establishment of image-guided optical navigation systems, enhancing the accuracy and safety of such interventions^3,17^.

As a result, image-based navigation is increasingly used to improve the drilling accuracy in different surgical applications, such as pedicle screw placement in spine surgery^17,18^, total shoulder arthroplasty and fracture fixation in orthopedic surgery^3,19,20^, implant socket drilling in dental surgery^21^, or zygomatic implant placement in oral and maxillofacial surgery^22^. A systematic review showed that reported rates of pedicle screw misplacement ranged from 6–31% for the freehand technique, while with conventional navigation systems (CNS) misplacement rates were reduced to 0–11%^17^.

While CNSs have proven to increase accuracy, they are limited by the cognitive challenge of integrating 2D imagery with 3D spatial understanding and issues related to hand-eye coordination^23,24^. This cognitive discontinuity requires surgeons to mentally reconstruct a 3D surgical space from multiple 2D images, a task that relies heavily on their spatial cognitive abilities^24^. Furthermore, hand-eye coordination problems may occur if the direction of movement shown on the monitor does not match the actual movement of the surgeon’s hands. This could place a significant cognitive burden on the surgeon who have to constantly shift their attention between the monitor and the surgical field to check anatomical locations and landmarks and synchronize their hand movements with what they see^23^. These limitations compromise the procedure efficiency and increase the likelihood of surgical complications by disrupting the spatial orientation of the surgeon and attention to critical anatomical details^25,26^.

Augmented Reality (AR), particularly through head-mounted displays (HMDs) provides a solution by integrating critical data and guidance direct into the surgeon's field of view^27^, thus potentially improving the surgical navigation by enhancing the accuracy^23,28^ and reducing the cognitive workload^29^. Indeed, studies on AR-based navigation systems (ARNS) have been able to demonstrate their technical feasibility in trajectory drilling. They mainly focused on the specific scenario, for example, pedicle screw placement^27,28,30^, fracture fixation^31,32^, total shoulder arthroplasty^33^, or dental implant placement^34^, although the latter is not generalizable due to the use of a dental contra-angle handpiece instead of a surgical drill and very short trajectories. In addition, some studies have demonstrated the clinical feasibility of ARNS, particularly in the context of pedicle screw placement, either comparing it to a freehand approach or evaluating the results using a clinical score^35,36^, or in pilot studies for dental placement^37^. However, statements about the actual accuracy or generalizability of the navigation system’s application are constrained by the studies’ methodologies like limited scope of application, limited number of participants, often lacking comparisons to CNS with optical tracking techniques, and adoption of tracked sleeve, which impedes real-time depth information. Only a very limited number of studies have directly compared the surgical drilling performance of ARNS with that of CNS (Table 1), none in a randomized controlled trial (RCT).

**Table 1:** Overview of studies comparing ARNS to CNS

Study	Year	Type	HMD	Registration	Tracking	Visualization	Error			Task	Subjects	Tracked Instrument	Anatomy	Model	Evaluation
							Translational (Mean ± Sd) (mm)	Angular (Mean ± Sd) (◦)	Depth						
Mueller et al.^27^	2020	Expl.	HL 1	IBR	MT (ARNS) OT (CNS)	Superimposition	3.4 ± 1.6^a^ (ARNS) 3.2 ± 2.0^a^ (CNS)	4.3 ± 2.3 (ARNS) 3.5 ± 1.4 (CNS)	None	Pre-drilling +insertion of K-wire	1 Surgeon (expert)	Sleeve	Complex anatomy (spine)	3 Cadaver Specimens	CT
Wolf et al.^30^	2023	Expl.	HL 2	PPR	OT (ARNS/CNS)	Superimposition, Virtual Twin, Sectional Views, Target Cross, Peripheral Rings	None	Expert/ Novices (median) 1.7/1.8 (CNS) 1.1/0.8 (Target Cross) 2.0/2.0 (Peripheral Rings) 1.7/1.4 (Overlay) 0.9/1.2 (Virtual Twin) 1.2/1.2 (Sectional Views)	None	Drilling holes	14 (10 novices + 4 experts)	Sleeve	Complex anatomy (spine)	Phantom	OT
This study	2024	XO RCT	HL 2	IBR	OT (ARNS/CNS)	Virtual Twin	1.11 ± 0.47^d^ (ARNS), 1.04 ± 0.47^d^ (CNS). 0.95 ± 0.42^a^ (ARNS), 0.98 ± 0.46^a^ (CNS). 0.93 ± 0.41^b^ (ARNS), 0.97 ± 0.45^b^ (CNS). 1.00 ± 0.52^c^ (ARNS), 0.92 ± 0.51^c^ (CNS).	1.11 ± 0.61 (ARNS) 0.73 ± 0.36 (CNS)	1.27 ± 1.59 (ARNS) 0.52 ± 0.82 (CNS)	Drilling holes	36 (12 students, 12 surgeons, 12 engineers)	Drill	Universal	Foam block	CT

*Expl.* Exploratory, *HL* HoloLens, *IBR* Image-based Registration, *MT* Mark-based Tracking, *OT* Optical Tracking, *PPR* Paired Point Registration, *XO RCT* Crossover Randomized Controlled Trial.

^a^Translational deviation at entry points in 3D Euclidean distance.

^b^Projected translational deviation at the entry point: shortest distance from the entry point of the conducted trajectory to the planned trajectory.

^c^Projected translational deviation at the endpoint: shortest distance from the end point of the conducted trajectory to the planned trajectory.

^d^Maximum projected translational deviation: the larger value of the projected translational deviations at the entry and end points of the conducted trajectory.

**Fig. 1:**
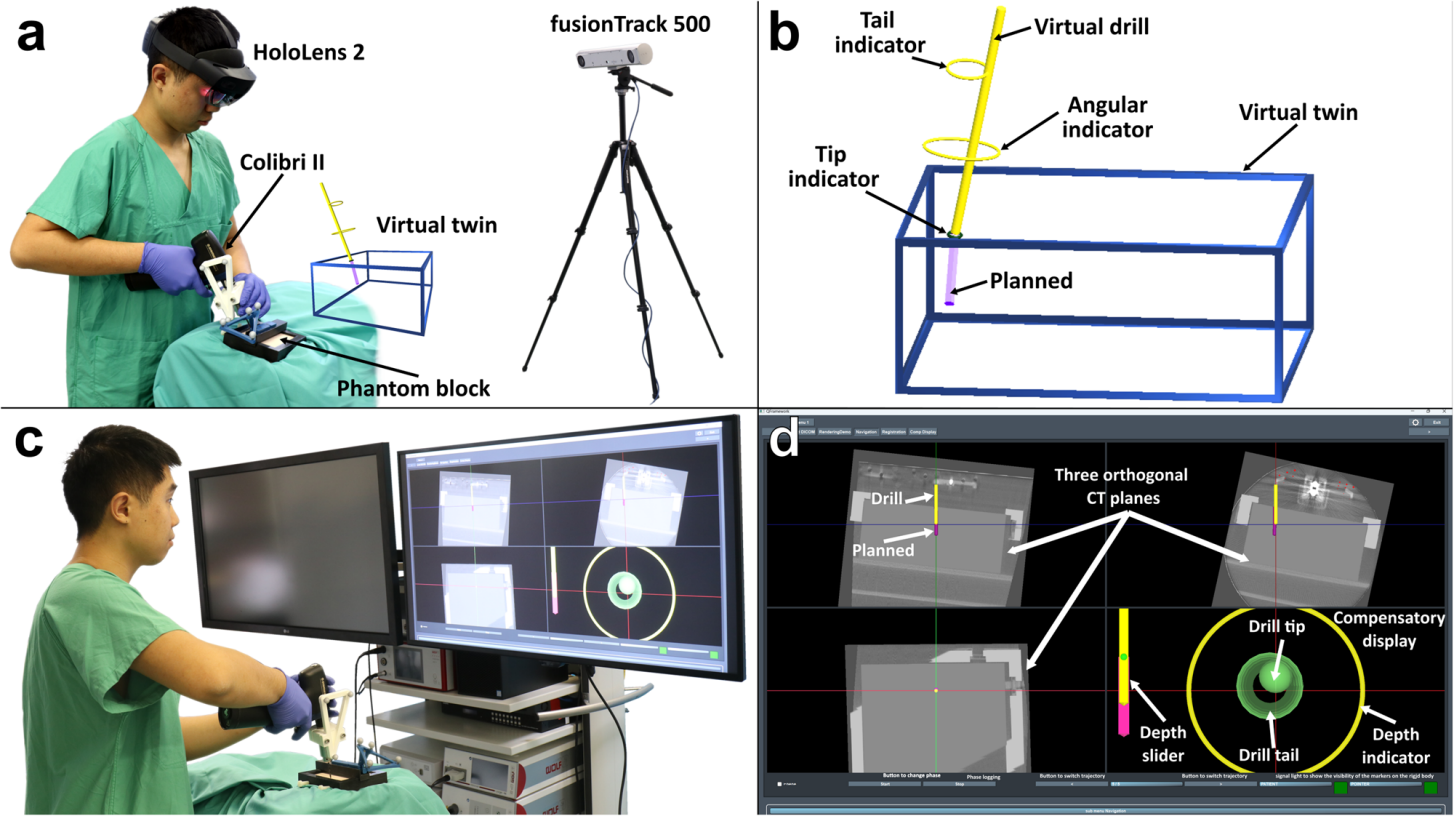
Experimental setup. **a** ARNS using HoloLens 2 with virtual twin representations of the block (blue) and drill (yellow) are displayed next to the phantom. **b** ARNS presented a virtual block with a blue boundary, accompanied by a drill and guidance. The guidance consisted of two tori for the translational deviation of the drill tip (tip indicator) and tail (tail indicator) from the planned trajectory, while a middle torus in ellipse shows the angular deviation. The colors of all indicators turn green if the translational and angular errors are within 1mm and 1°. The virtual drill turns green from yellow when it reaches the planned depth and red if deeper (>1 mm). **c** The CNS setting displayed drilling navigation on an external 2D monitor (right). The optical tracker is not shown. **d** The graphical user interface of CNS displays three sub-windows with orthogonal CT planes of the phantom block, illustrating the spatial alignment of the drill with the trajectory. A compensatory view in the lower right sub-window provided a transverse trajectory perspective that enhanced the accuracy of the drill alignment. The circle (represents drill tip) and middle ring (tail) change from yellow to green when translational error is ≤ 1 mm. A depth slider to the left visualizes the current depth (yellow) relative to the planned depth (purple). The slider and the outer ring change from yellow to green at the planned depth (0–1 mm) and turn red if it exceeds that depth by >1 mm.

Given these limitations, our study aimed to comprehensively compare the accuracy and efficiency of ARNS and CNS in drilling trajectories in a crossover RCT design (Fig. 1). The primary endpoint was the maximum projected translational deviation between the planned and performed trajectories, and the secondary endpoints were the translational deviation at the entry point in 3D Euclidean distance, projected translational deviation at the entry and end points, angular deviation, depth deviation, time, workload using NASA-TLX, and overall user experience using the System Usability Scale (SUS).

## Results

### Cohort

36 participants (10 female and 26 male) from three groups (surgeons, medical/dental students, and engineers) each with 12 participants were successfully included. The mean age of participants was 31.2 ± 9.6 (mean ± s.d.; range: 20–59). The average clinical experience of surgeons was 11.0 ± 8.8 years, the average clinical semesters of medical/dental students were 3.7 ± 2.1 (1.8 ± 1.0 years) and the average work experience of engineers was 5.5 ± 9.7 years. Eleven participants had experience with CNS, most of whom were surgeons. Nine participants had experience with AR-HMD, most of whom were engineers (Table 2).

**Table 2:** Characteristics of the cohort

Parameter		Surgeon (*n* = 12)	Student (*n* = 12)	Engineer (*n* = 12)	Total (*n* = 36)
Sex	female	1 (8.3%)	5 (41.7%)	4 (33.3%)	10 (27.8%)
	male	11 (91.7%)	7 (58.3%)	8 (66.7%)	26 (72.2%)
Age	mean (SD)	37.8 (8.6)	24.6 (3.3)	31.2 (10.6)	31.2 (9.6)
	range	29–55	20–30	24–59	20–59
Clinical Study / Work Experience (Years)	mean (SD)	11.0 (8.8)	1.8 (1.0)	5.5 (9.7)	n.a.
	range	2.0–30.0	0.5–3.5	0.0–33.0	n.a.
Previous Experience with CNS	no	3 (25.0%)	12 (100.0%)	9 (75.0%)	24 (66.7%)
	yes	9 (75.0%)	0 (0.0%)	3 (25.0%)	12 (33.3%)
Previous Experience with AR	no	9 (75.0%)	10 (83.3%)	6 (50.0%)	25 (69.4%)
	yes	3 (25.0%)	2 (16.7%)	6 (50.0%)	11 (30.6%)
Preferred Navigation System	CNS	5 (41.7%)	2 (16.7%)	2 (16.7%)	9 (25.0%)
	ARNS	7 (58.3%)	10 (83.3%)	10 (83.3%)	27 (75.0%)

### Drilling accuracy and efficiency

**Fig. 2:**
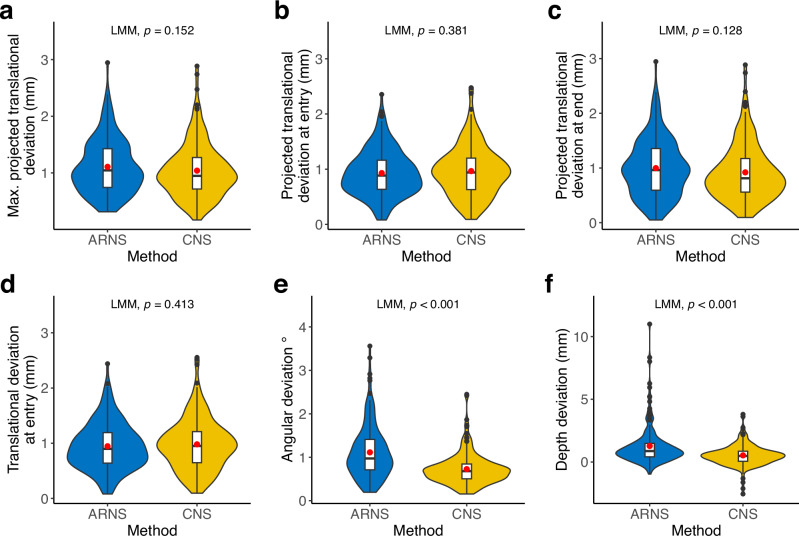
Comparison of accuracy by method. **a**–**f** Comparison of accuracy metrics between ARNS (blue) and CNS (yellow) methods (x-axis). Each violin plot (colored) includes a boxplot (white) with a red point marking the mean value. The black points are outliers. The results represent the average evaluations of the segmented meshes by two independent investigators. *P* values are from the corresponding LMM model. **a** Maximum projected translational deviation in mm (y-axis). **b** Projected translational deviation at the entry points in mm (y-axis). **c** Projected translational deviation at the endpoints in mm (y-axis). **d** Translational deviation in 3D Euclidean distance at entry points in mm (y-axis). **e** Angular deviation in degree (y-axis). **f** Depth deviation in mm (y-axis).

In the postoperative CBCT scans, all 360 trajectories were evaluated (without the 72 for familiarization). ARNS and CNS demonstrated comparable accuracy in translational alignment. The primary endpoint, the maximum projected translational deviation between the executed and planned trajectories, showed no significant difference between ARNS with 1.11 ± 0.47 mm and CNS with 1.04 ± 0.47 mm (LMM, *p* = 0.152; Fig. 2a). Both systems also exhibited similar projected accuracy at entry and endpoints with deviation at entry points for ARNS and CNS being 0.93 ± 0.41 mm and 0.97 ± 0.45 mm (LMM, *p* = 0.381; Fig. 2b) and at endpoints 1.00 ± 0.52 mm for ARNS compared to 0.92 ± 0.51 mm for CNS (LMM, *p* = 0.128; Fig. 2c). Additionally, the deviations remained comparable with the translational deviation at the entry point measured in 3D Euclidean distance. ARNS showed 0.95 ± 0.42 mm and CNS 0.98 ± 0.46 mm, again with no significant difference (LMM, *p* = 0.413; Fig. 2d).

**Fig. 3:**
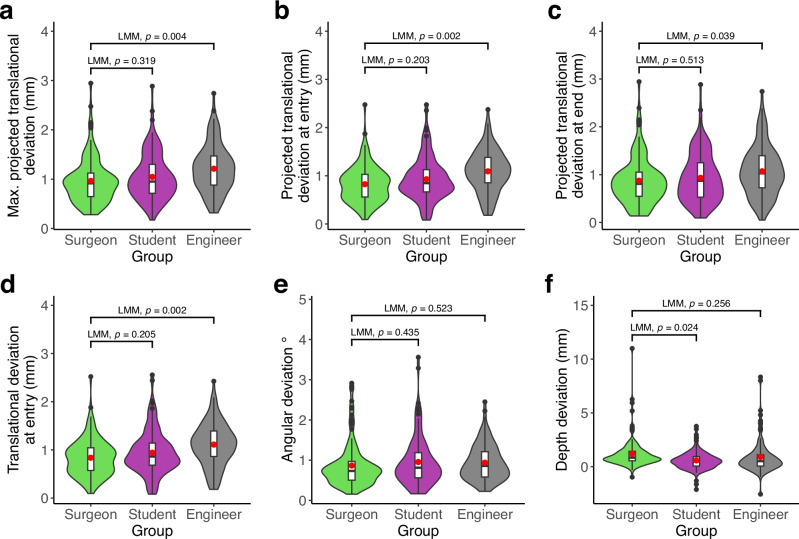
Comparison of accuracy by profession. **a**–**f** Comparison of accuracy metrics between the surgeon (green), student (purple) and engineer (gray) groups (x-axis). Each violin plot (colored) includes a boxplot (white) with a red point marking the mean value. The black points are outliers. The results represent the average evaluations of the segmented meshes by two independent investigators. *P* values are from the corresponding LMM model. **a** Maximum projected translational deviation in mm (y-axis). **b** Projected translational deviation at the entry points in mm (y-axis). **c** Projected translational deviation at the endpoints in mm (y-axis). **d** Translational deviation in 3D Euclidean distance at entry points in mm (y-axis). (**e**) Angular deviation in degree (y-axis). **f** Depth deviation in mm (y-axis).

**Fig. 4:**
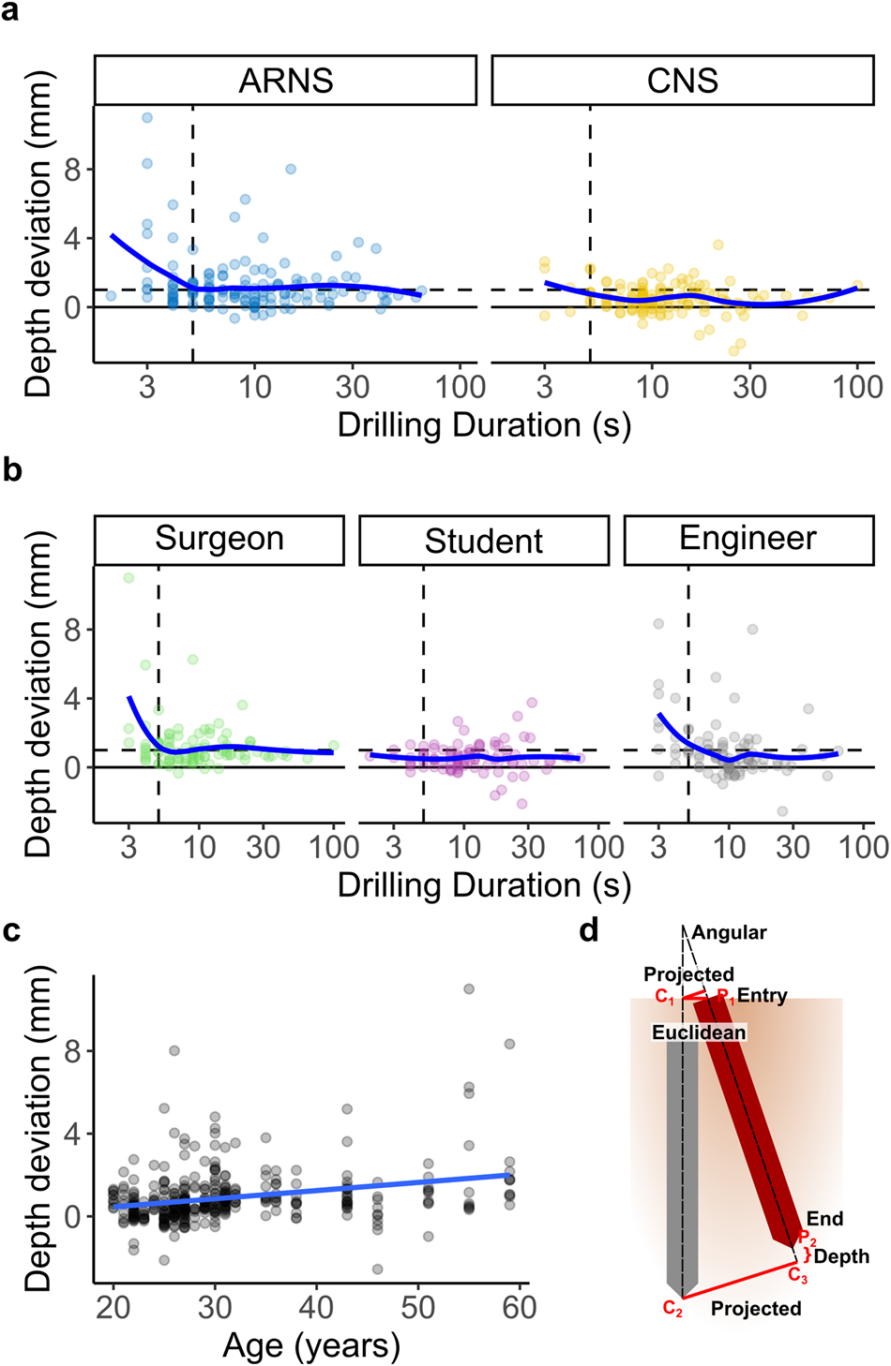
Additional investigation of depth deviation. **a**, **b** The relationship between drilling time in seconds (x-axis) and depth deviation in mm (y-axis); the blue line represents the LOESS (locally estimated scatterplot smoothing) function, while the dots indicate individual measurements. The vertical dashed line marks the drilling time of 5 seconds, while the horizontal dashed line marks the depth deviation of 1 mm and the solid line marks the depth deviation of 0 mm. **a** ARNS (left) and CNS (right), individual measurement points are shown in blue (ARNS) and yellow (CNS). **b** Surgeons (left), Students (middle), and Engineers (right), individual measurement points are shown in green (Surgeons), purple (Students), gray (Engineers). **c** Relationship between participants' age in years (x-axis) and their drilling depth deviation in mm (y-axis); the blue line represents the linear model (LM) function, and the dots indicate individual measurements. **d** Visualization showing the calculation of the accuracy metrics used. The planned drill hole (25 mm long) is shown in red, the conducted drill hole is shown in gray. Angular deviation was calculated as the angle between the two vectors (dashed lines). Projected translational deviation at entry (red line, C_1_ to planned vector) and end (red line, C_2_ to C_3_/planned vector) as orthogonal distance. The maximum projected translational deviation (primary endpoints) is the larger value of the two projected translational errors. Euclidean deviation (red line, C_1_ to P_1_) on the surface and depth deviation (red curly brackets, P_2_ to C_3_).

While for the other secondary endpoints, the angular deviation was 1.11 ± 0.61° in the ARNS, significantly higher than the CNS 0.73 ± 0.36 ° (LMM, *p* < 0.001; Fig. 2e). Similarly, the depth deviation for ARNS is 1.27 ± 1.59 mm, significantly higher than the CNS with 0.52 ± 0.82 mm (LMM, *p* < 0.001; Fig. 2f). Interestingly, the subgroup analysis showed slight differences between surgeons, students and engineers. According to the LMMs, the engineers were worse at translational deviations and the students were better at depth deviation (LMM, *p* < 0.05; Fig. 3). Depth deviation outliers occur primarily in ARNS and rapid drilling (Fig. 4a), mostly by surgeons and engineers (Fig. 4b). Furthermore, we observed a correlation between age and depth deviation (LMM, *p* = 0.010, Fig. 4c). An additional analysis of the primary endpoint and secondary endpoints by profession is available in the Supplementary Information (Supplementary Table 1).

**Fig. 5:**
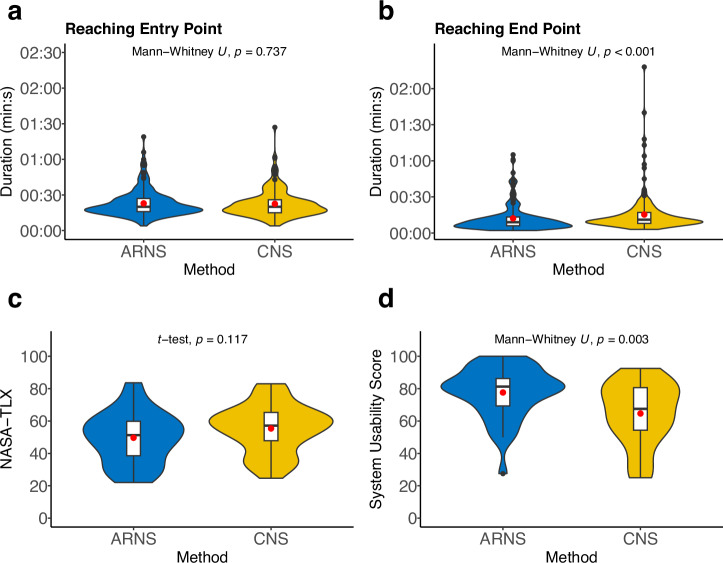
Comparison of efficiency. **a**–**d** Comparison of time and subjective scales between ARNS (blue) and CNS (yellow) methods (x-axis). Each violin plot (colored) includes a boxplot (white) with a red point marking the mean value. The black points are outliers. **a** Results of the time to find the entry point of ARNS and CNS methods (y-axis). **b** Results of the time to drill until reaching the planned depth (y-axis). **c** Results of subjective workload by NASA-TLX, where the higher values correspond to greater workload (y-axis). **d** Results of subjective assessments of overall all user experience by System Usability Score, where higher values correspond to greater user experience (y-axis).

Nine out of 720 time periods of drilling were excluded from the analysis due to invalid logging. There was no significant difference in the time to locate the entry point between ARNS and CNS, recorded at 22.9 ± 11.4 s and 22.5 ± 11.6 s, respectively (Mann–Whitney *U* test, *p* = 0.737; Fig. 5a). However, there was a notable difference in drilling completion time with ARNS taking 12.2 ± 11.2 s and with CNS taking 15.3 ± 16.0 s (Mann–Whitney *U* test, *p* < 0.001; Fig. 5b). There was no significant difference in NASA-TLX workload score between ARNS 49.67 ± 15.76 and CNS 55.40 ± 14.87, with a mean difference of 5.73 (Unpaired *t*-test, *p* = 0.117) (Fig. 5c; Supplementary Table 1). However, the results of the SUS concerning the overall user experience of ARNS with 77.64 ± 15.78 was significantly higher than the CNS with 64.65 ± 19.17 (Mann–Whitney *U* test, *p* = 0.003; Fig. 5d).

### Questionnaires

The Likert-type questions (scored from 1 to 4, with 1 indicating strong disagreement and 4 indicating strong agreement) revealed that ARNS was generally preferred over CNS. Specifically, ARNS was perceived as better than CNS at finding the entry point (3.4 vs. 2.8), setting the orientation (3.4 vs. 2.9), being easy to use (3.4 vs. 2.9), being beneficial for drilling (3.5 vs. 2.9), integrating (3.4 vs. 2.8), and being intuitive (3.4 vs. 2.8), drilling more accurately (2.9 vs. 2.5) compared to CNS. However, ARNS did not show clear advantages in safety perception (3.2 vs. 2.8), systematic complexity (1.6 vs. 1.9), drilling interference (1.5 vs. 1.8), need for technical assistance (2.1 vs. 2.1), and need for learning (2.0 vs. 2.4), but these did not affect the significant advantages of ARNS in other key performance indicators and overall better performance than CNS (Table 3).

**Table 3:** Likert Questionnaire

#	Likert questions	AR method (*n* = 36)	Conventional method (*n* = 36)	Difference	*p*
1.	The (AR-based | conventional) navigation system interfered with my drilling trajectory.	1.5 (0.7)	1.8 (0.9)	−0.3	0.189
2.	With the (AR-based conventional) navigation system, it is easy to find the starting point.	3.4 (0.7)	2.8 (1.0)	0.6	0.007
3.	With the (AR-based conventional) navigation system, it is easy to set the orientation.	3.4 (0.8)	2.9 (0.8)	0.5	0.012
4.	The (AR-based | conventional) navigation system is easy to use.	3.4 (0.8)	2.9 (1.0)	0.5	0.017
5.	I felt safe using the (AR-based | conventional) navigation system.	3.2 (0.7)	2.8 (0.9)	0.4	0.073
6.	The (AR-based | conventional) navigation system is good for drilling trajectories.	3.5 (0.7)	2.9 (0.7)	0.6	0.001
7.	I find the (AR-based | conventional) navigation system unnecessarily complex.	1.6 (0.8)	1.9 (0.9)	−0.3	0.180
8.	I think I would need the assistance of a technical person to use the (AR-based | conventional) navigation system.	2.1 (0.9)	2.1 (1.0)	0.0	0.958
9.	I think that the various functions in the (AR-based | conventional) navigation system were well integrated.	3.4 (0.8)	2.8 (0.8)	0.6	0.001
10.	I still have a lot to learn before I could use the (AR-based | conventional) navigation system.	2.0 (0.8)	2.4 (1.0)	−0.4	0.124
11.	I found the (AR-based | conventional) navigation system intuitive.	3.4 (0.7)	2.8 (0.9)	0.6	<0.001
12.	I drilled the trajectories accurately with the (AR-based | conventional) navigation system.	2.9 (0.7)	2.5 (0.8)	0.4	0.035

In addition, different positive and negative feedback about the ARNS and CNS were provided (Table 4). Correspondingly, in total 27 (75%) participants preferred the ARNS, while 9 (25%) participants preferred the CNS (Table 2). An additional analysis of the questionnaires by profession is available in the Supplementary Information (Supplementary Tables 1 and 2).

**Table 4:** Open questions summarized

Method	Positive^a^	Negative^a^
ARNS	• Easy to find the start point • Easy to align/orient the instrument • Drilling faster and safer • Easy to learn/shorter learning curve • Intuitive • All information in one display • Well-integrated guide functions • Larger display field • Smoother and more stable feedback	• Dependent on HMD • Display of frames is sometimes interrupted • Hard to algin instrument to the center of the ring • The design of the functions is complex • The blue frame occluded the view to see the drilling depth • The color changing from yellow to green is ambiguous • Hard to control depth • Concentration on 3D hologram instead of patient • Motion sickness • Need of extra instruction
CNS	• No reliance on HMDs • Direct and easy to use • Multiple perspectives to observe and check while drilling • Depth control is better with the depth slider • More degrees of freedom in alignment	• Difficult to find the starting point • Need to switch attention between four windows • The display view changes along the tip of the instrument, which is annoying to the user • Hand eye coordination problem, need to switch attention from patient to screen constantly • Longer learning curve

^a^Positive and negative feedback from surgeons (green rectangle), students (purple triangle), and engineers (gray diamond).

## Discussion

To our knowledge, this is the first crossover RCT dedicated to systematically comparing ARNS and CNS to evaluate the accuracy and efficiency of drilling trajectories. The principal findings revealed no significant difference in translational deviation. However, CNS demonstrated better performance in angle and depth deviation compared to ARNS, although these generally did not have significant clinical implications, except for cases involving outliers. For instance, during the placement of pedicle screws, the differences between two systems should not result in a deviation exceeding 2 mm, thus allowing both systems to be categorized under the same classification^38^. Notably, drilling time was significantly faster with ARNS, but without clinical relevance. In practical terms, ARNS and CNS were effectively comparable in their drilling performance. NASA-TLX workload was comparable between the two methods. While ARNS markedly outperformed CNS in terms of overall user experience. In general, 75% of the participants preferred the ARNS, while only 25% of the participants preferred the CNS.

The need for such a confirmatory study arises from the limitations observed in previous research, which employed exploratory designs^27,28,30–33,39^, including limited participants and/or lack of control groups, thereby compromising the reliability and validity^27,28,31,33,39^. On the contrary, our study adopted a confirmatory design, with pre-registration of a study protocol, properly powered based on sample size calculation and predefined effects, ensuring reliable and valid results in the systematic comparison of an ARNS with a CNS as control. This rigorous approach is key to definitively assessing the accuracy and efficiency of ARNS in trajectory drilling and providing solid insights into the field.

In the past, drilling guided by CNS with optical tracking has demonstrated good accuracy in many surgical fields^17^, including pedicle screw placement^40^ or zygomatic implant surgery^41^. According to the findings of various studies, the range of translational and angular deviation has been reported to be between 1.27 and 6.43 mm and between 2.68 and 3.09° for the CNS^22,40–42^. Our CNS evaluated by post-CT showed good results among the above-mentioned studies, thereby making it a valid control. Similarly, the feasibility of ARNS was demonstrated by a number of studies, yet without a comparison to CNS. The range of translational and angular deviation was reported to be between 1.4 mm and 2.77 mm, and between 3.0° and 3.8°, respectively^28,32,33,39^. In contrast, our ARNS obtained results in translational deviation of 0.95 ± 0.42 mm, which together with angular deviation of 1.11 ± 0.61° outperformed the ARNS in the above-mentioned feasibility studies.

Nevertheless, only two other studies beside ours compared ARNS with CNS for surgical drilling (Table 1). Yet, this is necessary to consider the individual settings and factors (registration, tracking system used, drill skiving, type of visualization, etc.) that inevitably occur in any study. Mueller et al. had no significant difference between ARNS (3.4 ± 1.6 mm/4.3 ± 2.3°) and CNS (3.2 ± 2.0 mm/3.5 ± 1.4°). However, the study of Mueller et al. was limited by the low accuracy of marker-based tracking (>2.0 mm/>2.0°) in ARNS and the limited number of participants (1 experienced surgeon)^27^. Wolf et al. claimed that the ARNS (virtual twin: median angular deviation 0.9/1.2°) outperformed the CNS (median: 1.7/1.8°) for the two study groups (experts/novices). In contrast, our ARNS and CNS achieved better accuracy with a median angular deviation of 0.97° and 0.68°, respectively. Although the results of Wolf et al. provide good insight into optimal ARNS visualization, the validity of the results is limited because their evaluation was based solely on tracking data (4% of measurements had to be excluded due to incompleteness by Wolf et al.^30^). The evaluation of tracking data instead of postoperative CTs has potential additional errors^30^. This may compromise the translation of findings into real-world clinical settings. In addition, only angular deviations were evaluated^30^, lacking depth or translational information due to the exclusive use of a tracked sleeve, which may be to some extent depending on the surgical scenario.

Our ARNS benefits from the incorporating the virtual twin and state-of-the-art imaging-based registration (IBR) and optical tracking technique. Many studies used procedural registration methods to map the image data to the patient, such as Paired Point Registration (PPR)^30,31^ and Iterative Closest Point (ICP)^32,33,39^. However, these methods are prone to human error and inaccuracy, which reduces the overall accuracy during navigation. In contrast, other studies, like ours, used IBR, which is widely used in surgical navigation systems^43,44^. Compared to procedural registration such as ICP or PPR, IBR has been shown to be more accurate, improving the accuracy of both of our methods, but at the cost of additional radiation exposure^45^. Apart from registration, tracking itself is another possible source of inaccuracy. In contrast to optical tracking, 2D fiducial marker-based tracking has demonstrated a wide range of root mean square error (RMSE), ranging from 0.87 mm to more than 10 mm^46^, although it is commonly used in studies^27,33,39^. However, most of the optical tracking cameras available on the market achieved an accuracy of RMSE less than 0.5 mm, especially for the fusionTrack 500 in our study, the accuracy in RMSE of 0.09 mm (for up to 2 meters) is outstanding among them^47^.

Although superimposition is often perceived as the intuitive way of AR visualization in healthcare applications and used in many studies, it has many drawbacks, especially when using OST HMDs^48^. The holographic overlay can be prone to focus rivalry and vergence-accommodation conflict, which can disrupt the surgeon's depth perception, distort the observation of important anatomical structures and increase cognitive load. Moreover, superimposition introduces additional errors in the registration between virtual and physical counterparts. In literature, the reported registration accuracy was in the range of 0.62 to 6.93 mm in translation, and the average angular accuracy was in the range of 1.32° to 6.80°^49^, which could have significant impacts on navigation accuracy in the end. Overall, this may subsequently lead to a reduction in accuracy^50,51^. However, our ARNS adopted “virtual twin” free from this registration error in OST, as everything takes place in virtual space. Correspondingly, Wolf et al. reported that this “superimposition” visualization was the most distracting of five possible ARNS visualization approaches, making it difficult for users to locate the tooltip. On the contrary, a virtual twin ARNS visualization performed better in terms of orientation and was rated higher in terms of overall user experience and cognitive load^30^.

Another source of error may be skiving, which may have occurred in the aforementioned studies. Skiving is the displacement of the drill during drilling caused by the geometry of the drill tip^52,53^. Skiving can be reduced by using a sharper drill tip^53^. Therefore, the tip was sharpened from 118° to 90° in this study. Furthermore, the solely navigated sleeve configuration in some studies could also account for the limited accuracy^27,28,30,39^, where the lack of real-time depth information and tolerance introduces error into the final results^54^. By using sleeves, tolerance errors and increased friction from sleeves could compromise accuracy^54^. The use of sleeves raises temperatures, which could potentially result in thermal osteonecrosis^11,55^. If the sleeve is tracked instead of the drill, real-time depth information is not provided by the navigation system. If the sleeve does not limit the allowable depth, this could result in nerve damage in spine and dental implant surgery and cortical bone penetration in orthopedic surgery^17,56,57^.

Furthermore, due to limitations of the trial design in the aforementioned studies, confounding factors were not addressed. Therefore, the observed differences between ARNS and CNS could be caused by bias. In contrast, our RCT addressing confounding shows that there is no significant difference in translation deviation. However, we found significant differences in the mean angular deviation of the ARNS compared to the CNS (1.11° vs. 0.73°). In terms of depth, our ARNS were found to over drill by an average of 1.27 mm compared to the CNS with an average of 0.52 mm. Although the ARNS results showed a mean difference in the sub-millimeter/-degree range for depth deviation (0.75 mm) and angle deviation (0.39°) compared to CNS, from a clinical perspective and excluding outliers, this difference would not be clinically relevant in the vast majority of surgical scenarios. For example, in pedicle screw placement surgery, a breach of less than 2 mm is considered acceptable^38^.

Increased deviations for target orientation and target depth with ARNS compared to CNS may have been caused by differences in the graphical user interface (GUI). GUI differences are the result of the different display types (i.e., external 2D view, first-person 3D view, compensatory top-down display), which may also result in different scaling of the navigation information. However, we did not find any significant differences in translation errors. Yet, we found other possible explanations for the observed differences. For example, the depth guidance in ARNS was based on the spatial position of the virtual drill and the planned position, as well as a color transition from yellow to green (correct depth) and then to red (too deep). Interestingly, four participants reported that depending on the positioning of the HL2 on the head, the colors were distorted (chromatic aberration)^58^. This caused yellow objects to appear green, making it difficult for the study participants to judge when the correct target depth had been reached, and may explain some of the outliers in depth for the ARNS. This is a technical limitation of HL2, which only became evident after the inclusion of a larger number of participants.

Although stereoscopic visualization is seen as an advantage in hand-eye coordination, it could bring a limitation in this task. In the compensatory display of the CNS, the angular deviation was seen from top-down, so that deviations in all spatial directions were detected immediately. In the ARNS, however, the view was almost always from one direction depending on the position of the user. In addition, the stereoscopic vision of the participants (despite passing Lang-Stereotest II) may have introduced a further error. Both could have been reasons for the reduced angular deviation.

In the subgroup analysis in accuracy, there was no significant difference between student and surgeon groups (Fig. 3a–e, despite f). But the engineer group underperformed significantly in the translational deviation compared with other two groups (Fig. 3a–d). One reason may be the superior hand-eye coordination resulting from the accumulated experience of surgical training for the surgeons. Interestingly, the students performed best at depth deviation, probably due to the younger age of this group with faster motor reaction time (LMM adjusted for age, age *p* = 0.010; Fig. 4c). This finding is consistent with studies of motor reaction time and age in the literature^59^.

Nevertheless, these possible causes of sub-millimeter/-degree differences in angular and depth deviation between ARNS and CNS could probably be resolved in the future by optimizing the GUI and HMD hardware used, approaching the limits of the technical accuracy of the registration method and the optical tracking system. In summary, although the ARNS is slightly inferior to the CNS on angular and depth deviations, it is clinically close to the specifications of the CNS. Yet, ARNS was preferred more often and had better user experience regardless of professional background, probably due to its easy to understand and intuitive visualization. However, only 58.3% of the surgeons preferred ARNS while 41.7% preferred CNS. The reason for this was that all surgeons (*n* = 3) with no previous experience in CNS preferred the CNS. Among surgeons with previous experience in CNS (*n* = 9), 77.8% (*n* = 7) preferred the ARNS, while surgeons in general (including those without experience in surgical navigation) had mixed opinions. Furthermore, the average surgical experience of the surgeons who preferred ARNS to CNS was slightly higher (12.0 years) compared to those who preferred CNS (10.4 years).

The limitations of this study are that although we were able to demonstrate the technical performance of ARNS on a phantom in postoperative CTs and the benefit to usability, the potential benefit for a real-life scenario such as zygomatic implantation or pedicle screw placement needs to be demonstrated to account for real operating room settings where target surgical sites have complexed structure instead of a flat surface.

Finally, all studies, including ours, have conducted a superiority study. For clinical use, non-inferiority study (calculating confidence intervals instead of inference statistics) in particular could be one of the leading aspect for integration into the clinical workflow. In this regard, the findings and results of our study can be used for a first non-inferiority study in the future.

In this RCT we were able to for the first time provide compelling evidence that ARNS and CNS have comparable accuracy in translational error. The observed differences in angle and depth deviation are probably due to limited stereoscopic vision, hardware and setup limitations, and the design concept of the ARNS. In the future, these factors could be addressed by adapting the hardware and guidance. Nevertheless, the ARNS was preferred over the CNS by most participants and the majority of surgeons with previous navigation experience, with significant overall better user experience. Altogether, depending on the accuracy required, ARNS could be a viable and possible alternative to the use of CNS for guided trajectory drilling.

## Methods

**Fig. 6:**
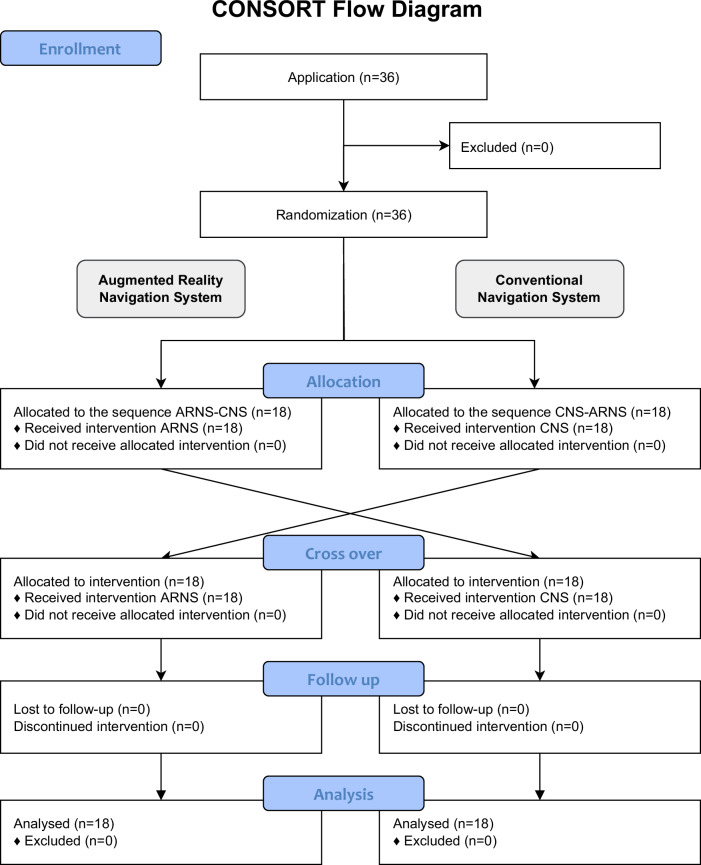
CONSORT flow diagram. CONSORT flow diagram illustrating the flow of participants from enrollment, through allocation, including cross-over, to follow-up and analysis. This diagram was created according to the requirements of the CONSORT reporting guidelines and modified for crossover studies.

36 subjects with different professional backgrounds and different levels of manual dexterity, who were right-handed and passed the Lang Stereotest II (assessment of spatial vision) (LANG-STEREOTEST AG, Switzerland), were recruited and performed trajectory drilling on a block phantom in a randomized cross-over order with ARNS and CNS (Fig. 6).

This study was approved by the local ethical commission of the University Hospital RWTH Aachen (EK 23-011; Chairman Prof. G. Schmalzing; approval date, 31.01.2023), has been registered in advance in the German Clinical Trial Register (DRKS00031357) with study protocol and followed the CONSORT 2010 guidelines and its extension for crossover studies^60,61^. Informed consent was obtained from all subjects involved in the study.

### Navigation systems

The ARNS system was developed through an interdisciplinary collaboration between medical engineers, medical informaticians, and multiple clinicians at a university hospital with extensive experience in surgical navigation systems. The ARNS software was programmed using C# in Unity (v2021.3.30f1) and was deployed on a HoloLens 2 (HL2) (Microsoft Corporation, Redmond, WA, USA). Visualization of a virtual twin was implemented as suggested by Wolff et al.^30^, with a holographic scene manually placed adjacent to the surgical field in a standardized manner (directly next to the phantom block on the opposite side of the patient; see Fig. 1a). The holographic scene displayed the phantom block as a 3D block outlined in blue, anchored stationary in the physical environment by the inherent function of the HL2. The virtual drill (yellow) was positioned relative to the virtual phantom block. This was based on the relative coordinates of the real drill and the real phantom block obtained in real time by the optical tracking system (Fig. 1a). Due to the use of relative coordinates, it was not necessary to transfer them to HL2 space. For user guidance, the translational and the depth deviation have been designed to be limited to 1 mm and the angle deviation to 1 °, respectively, which was considered safe and acceptable range^38^. To achieve this, three elements were present at the tip, middle, and tail of the drill, denoted by the lower torus, middle torus, and upper torus respectively. The adoption of the three tori was motivated by the study of Tu et al.^31^. The lower and upper tori adjusted their radius based on the shortest distance to the drill, with the inner radius having the exact diameter of the drill when the distance was ≤2 mm and changing accordingly when the distance was >2 mm. In addition, the color of the tori changed from yellow to green when the distance was ≤1 mm. The color of the virtual drill changed from yellow to green when the depth exceeded the target depth by 0–1 mm and to red >1 mm (Fig. 1b, Supplementary Movie 1). The rationale behind color feedback was motivated by color associations^62^.

The CNS software was based on an already developed software in C++ at the Chair of Medical Engineering (mediTEC). It was adjusted to meet the requirements for this study and to have similar guidance logic as the ARNS software. The CNS displayed the navigation information from the same optical tracking camera as the ARNS on an external 2D monitor (Fig. 1c). It featured a quad-display interface as many other CNSs used in OR, with three windows showing orthogonal multiplanar reconstructions (MPR) of cone-beam computed tomography (CBCT) scans of the phantom block, accompanied by a fourth compensatory display designed for precise trajectory correction. The MPR view depicted the real-time position of the drill (in yellow) to the preplanned trajectory (purple) and nearby structures of the phantom model, which, along with optical tracking, helped to precisely locate the entry point and perform accurate drilling. The compensatory display introduced a dual-circle visualization, a sphere, and a ring, representing the tip and the tail of the drill respectively to assist alignment of the drill to the trajectory in a top-down view. Beside, there was also a depth slider visualizing the current depth of the drill regarding the planned depth and the peripheral ring to indicate depth. This setup adopted the same color-coded feedback mechanisms as the ARNS: If either the central ring or the sphere has an alignment error ≤1 mm, it will turn from yellow to green. To ensure the depth accuracy, the depth indicator of the peripheral ring turned green to denote depth discrepancies within the 0–1 mm range and shifted to red for deviations >1 mm, thereby offering a clear, intuitive cue for real-time depth (Fig. 1d, Supplementary Movie 1).

The registration process between the tracker on the phantom and the CBCT data was identical for both systems. The CBCT scan was acquired using the Surgivisio imaging system (eCential Robotics, Gière, France), which performed auto-registration. The auto-registration was based on a fixed patient reference with an attached calibration phantom, as described in the literature^43,63^. The trajectories were planned in the coordinate system of the patient reference. A server software written in C++ sent the tracking data from an external optical tracking camera fusionTrack 500 (Atracsys LLC, Switzerland) via Wi-Fi to the ARNS and the CNS clients. The clients compute the spatial relationship transformation in exactly the same way and then display it in their respective visualization method.

### Sample size calculation

The sample size calculation was performed in R (version R4.3.1, www.r-project.org). The data needed to calculate the sample size was taken from a preliminary test with six participants. After excluding the first trajectory for practice, 60 drilled trajectories were evaluated, one of which was excluded due to technical failure of the system. The CNS achieved a projected maximum translational deviation of 1.45 ± 0.70 mm (mean ± SD) and that of the ARNS was 1.75 ± 1.28 mm. A simulation-based power analysis was performed to plan the number of cases for a linear mixed-effects model (LMM) with lmerTest Package^64^. This LMM assessed the maximal projected translational deviation, with navigation systems, starting navigation system (ARNS or CNS), and group (surgeon, student, engineer) as fixed effects, and sequence (the order to drill trajectories) and subjects as random effects. With significance level of α = 0.05, This resulted in 36 participants (including 4 for dropout) to meet the power of 80%.

### Trial

The study was conducted in a surgical setting using a phantom resembling cortical bone (PUR modeling board M330, Sika AG, Baar, Switzerland). This phantom was attached to a tracked 3D-printed frame, and the assembly was placed on a Vertebroplasty Trunk model (Sawbones Corporate, Washington, U.S.) situated on a table. The scenario consisted of six trajectories (25 mm length, 3 mm diameter), which were pre-planned and registered with the Surgivisio CBCT imaging system (eCential Robotics, Gières, France). The trajectories were angled at 10–15 degrees relative to the phantom’s upper surface normal. Each subject conducted the drilling with a tracked cordless handheld drill (Colibri II, DePuy Synthes, Indiana, US). To reduce skiving during drilling^53^, a 3.0 mm diameter drill bit was tipped at a 90° angle (Craftomat metal drill HSS-R Speed, BAHAG AG, Germany).

Each subject, stratified by profession, was randomly assigned to start with one of two navigation systems according to an urn randomization rule (sampling without replacement). The two blocks were balanced to 18 participants each, 6 in each professional group. The random allocation was planned and performed by B.P.

Participants received a brief introduction to the respective systems before proceeding with the drilling tasks. The first drilled hole was for practice and was not included in the later evaluation. Afterward, participants drilled five consecutive holes, with the system automatically displaying the next planned trajectory after each time the drill was removed from the drill hole. Thereby two time periods for each drill task were automatically recorded. The first period was from the start of each drill until the drill penetrated to a depth of 3 mm, which was referred to the time find the entry point. The second period was from the moment the depth reached 3 mm until the target depth (25 mm) was reached, which was referred to the drilling time. If the depth was not reached, the start time of the next trajectory was used as the end time of the current trajectory.

Upon completion of six times drilling with one navigation system, the subject completed a weighted NASA-TLX questionnaire to measure workload, SUS, and a Likert questionnaire for qualitative assessment. The other navigation system was then administered using the same procedure. Finally, the subject completed a final open-ended questionnaire to qualitatively compare the two systems.

### Evaluation

After the trial, all phantom blocks were scanned with the abovementioned CBCT scanner. To enhance the visibility of the drilling trajectories in the CT scan, rods 3D printed by a Prusa SLS SPEED were inserted. The rods' tips were designed to be flat, without the 1.5 mm long conical sharp tip (90°), to allow for maximum insertion into the drill hole, as debris could obstruct proper placement. The acquired CT scans were analyzed in 3D Slicer (v5.2.2). Two independent investigators (Y.L. and P.B.) evaluated the conducted trajectories before comparing them to the ground truth.

The rods were segmented using the SegmentEditorExtraEffects in 3D Slicer by outlining their path and then exported as OBJ meshes. If the discrepancy of the translational deviation was ≥ 0.5 mm or the angular deviation was ≥1.0° between two investigators, B.P. checked the discrepancy, then Y.L. and P.B. repeated the segmentations to reduce bias in segmentation. Supplementary Fig. 1a, b shows the conducted and planned trajectories, which were all displayed with the trajectory mesh for clear comparison.

Afterward, the models were automatically compared to the planned trajectories using a Python script (v3.6.1) with VTK packages. The calculations were performed as follows: ${P}_{1}$ and ${P}_{2}$ represented the entry point and the endpoints of the planned trajectory, whereas those of the conducted trajectory were denoted as ${C}_{1}$ and ${C}_{2}$. Entry points for both planned and conducted trajectories were where the mesh of the trajectories penetrated the block's upper surface. ${C}_{2}$ was identified as the furthest point along the central axis plus an additional 1.5 mm offset to account for the flat ends of the rods (Fig. 2d).

The maximum projected translational deviation (Fig. 2a) was the larger value of projected translational deviation at two ends (Fig. 2b, c), denoted as the minimum radius of the inclusion cylinder from the planned trajectory that covers the conducted trajectory. The projected translational deviation was the shortest distance from the conducted trajectory to the planned trajectory, which was the length of shortest distance from ${C}_{1}$ and ${C}_{2}$ to the central axis of the planned trajectory respectively (Fig. 2b, c). In addition, the translational deviation at the entry point was evaluated by 3D Euclidean distance between ${P}_{1}$ and ${C}_{1}$(Fig. 2d). The angular deviation in degrees was calculated by the angle between the planned and conducted trajectories, using the following function: ${Angular\; deviation}={\cos }^{-1}\left(\frac{\vec{A}* \vec{B}}{{||}\vec{A}{||}* {||}\vec{B}{||}}\right)$, where $\vec{A}$ and $\vec{B}$ were the vectors along the central axis of the planned and the conducted trajectory respectively (Fig. 2e). The depth deviation was calculated as the distance between ${P}_{2}$ and ${C}_{3}$, where ${C}_{3}$ is ${C}_{2}$ projected onto the planned trajectory axis (Fig. 2f).

### Statistical analysis

The statistical analysis was also performed in R. The translational deviation between the planned and conducted entry and endpoints, angular deviation, and depth, were evaluated with an LMM as described above in the sample size calculation. The perceived workload in NASA-TLX was evaluated by unpaired *t*-test. Time and SUS between systems were compared using Mann–Whitney *U* tests. Normal distribution was tested using the Shapiro–Wilk test. A *p* < 0.05 was considered significant.

## Supplementary information

Supplementary Information

Supplementary Movie 1

## Data Availability

The datasets used and/or analysed during the current study available from the corresponding author on reasonable request.

## Code availability

The underlying code for this study is not publicly available but may be made available to qualified researchers on reasonable request from the corresponding author.

## References

[1] Abumi, K., Itoh, H., Taneichi, H. & Kaneda, K. Transpedicular screw fixation for traumatic lesions of the middle and lower cervical spine: description of the techniques and preliminary report. *Clin. Spine Surg.* **7.1**, 19–28 (1994). 10.1097/00002517-199407010-00003 8186585

[2] Gefen, A. Optimizing the biomechanical compatibility of orthopedic screws for bone fracture fixation. *Med. Eng. Phys.* **24**, 337–347 (2002). 12052361 10.1016/s1350-4533(02)00027-9

[3] Briem, D. et al. 3D fluoroscopic navigated reaming of the glenoid for total shoulder arthroplasty (TSA). *Comput. Aided Surg. Off. J. Int. Soc. Comput. Aided Surg.* **16**, 93–99 (2011). 10.3109/10929088.2010.546076 21219118

[4] Mobbs, R. J., Sivabalan, P. & Li, J. Technique, challenges and indications for percutaneous pedicle screw fixation. *J. Clin. Neurosci. : Off. J. Neurosurg. Soc. Australas.* **18**, 741–749 (2011). 10.1016/j.jocn.2010.09.019 21514165

[5] Bonasia, D. E., Governale, G., Spolaore, S., Rossi, R. & Amendola, A. High tibial osteotomy. *Curr. Rev. Musculoskelet. Med.* **7**, 292–301 (2014). 25129702 10.1007/s12178-014-9234-y PMC4596221

[6] AlAzri, A., Mok, K., Chankowsky, J., Mullah, M. & Marcoux, J. Placement accuracy of external ventricular drain when comparing freehand insertion to neuronavigation guidance in severe traumatic brain injury. *Acta Neurochirur.* **159**, 1399–1411 (2017). 10.1007/s00701-017-3201-5 28555269

[7] Wang, G. et al. A fluoroscopy-based surgical navigation system for high tibial osteotomy. *THC* **13**, 469–483 (2005). 16340091

[8] Vogel, T. W., Dlouhy, B. J. & Howard, M. A. Don’t take the plunge: avoiding adverse events with cranial perforators. *J. Neurosurg.* **115**, 570–575 (2011). 21456895 10.3171/2011.3.JNS101310

[9] Chrcanovic, B. R., Pedrosa, A. R. & Neto Custódio, A. L. Zygomatic implants: a critical review of the surgical techniques. *Oral. Maxillofac. Surg.* **17**, 1–9 (2013). 22274763 10.1007/s10006-012-0316-y

[10] Gras, F. et al. Screw Placement for Acetabular Fractures Which Navigation Modality (2-Dimensional vs. 3-Dimensional) Should Be Used? An Experimental Study. *J. Orthop. Trauma* **26**, 466–473 (2012). 22357092 10.1097/BOT.0b013e318234d443

[11] Pandey, R. K. & Panda, S. S. Drilling of bone: a comprehensive review. *J. Clin. Orthop. Trauma* **4**, 15–30 (2013). 26403771 10.1016/j.jcot.2013.01.002 PMC3880511

[12] Jamil, M. et al. Comprehensive analysis on orthopedic drilling: a state-of-the-art review. *Proc. Inst. Mech. Eng. H J. Eng. Med.* **234**, 537–561 (2020). 10.1177/0954411920911283 32186229

[13] Brioschi, V., Cook, J. & Arthurs, G. I. Can a surgeon drill accurately at a specified angle. *Vet. Rec. Open* **3**, e000172 (2016). 27547423 10.1136/vetreco-2016-000172 PMC4964160

[14] Nakamura, S., Matsuda, K., Arai, N., Wakimoto, N. & Matsushita, T. Mini-incision posterior approach for total hip arthroplasty. *Int. Orthop.* **28**, 214–217 (2004). 15168084 10.1007/s00264-004-0570-1 PMC3456936

[15] Manbachi, A., Cobbold, R. S. C. & Ginsberg, H. J. Guided pedicle screw insertion: techniques and training. *Spine J. Off. J. North Am. Spine Soc.* **14**, 165–179 (2014). 10.1016/j.spinee.2013.03.029 23623511

[16] Clement, H., Heidari, N., Grechenig, W., Weinberg, A. M. & Pichler, W. Drilling, not a benign procedure: laboratory simulation of true drilling depth. *Injury* **43**, 950–952 (2012). 22177726 10.1016/j.injury.2011.11.017

[17] Gelalis, I. D. et al. Accuracy of pedicle screw placement: a systematic review of prospective in vivo studies comparing free hand, fluoroscopy guidance and navigation techniques. *Eur. Spine J.* **21**, 247–255 (2012). 21901328 10.1007/s00586-011-2011-3 PMC3265579

[18] Tarawneh, A. M., Haleem, S., D’Aquino, D. & Quraishi, N. The comparative accuracy and safety of fluoroscopic and navigation-based techniques in cervical pedicle screw fixation: systematic review and meta-analysis. *J. Neurosurg. Spine* 1–8 10.3171/2020.11.SPINE201877 (2021). 10.3171/2020.11.SPINE201877 34144517

[19] Verborgt, O. et al. Accuracy of placement of the glenoid component in reversed shoulder arthroplasty with and without navigation. *J. Shoulder Elb. Surg.* **20**, 21–26 (2011). 10.1016/j.jse.2010.07.014 21134663

[20] Sebaaly, A., Jouffroy, P., Emmanuel Moreau, P., Rodaix, C. & Riouallon, G. Intraoperative cone beam tomography and navigation for displaced acetabular fractures: a comparative study. *J. Orthop. Trauma* **32**, 612–616 (2018). 30299379 10.1097/BOT.0000000000001324

[21] Hoffmann, J., Westendorff, C., Gomez-Roman, G. & Reinert, S. Accuracy of navigation-guided socket drilling before implant installation compared to the conventional free-hand method in a synthetic edentulous lower jaw model. *Clin. Oral. Implants Res.* **16**, 609–614 (2005). 16164469 10.1111/j.1600-0501.2005.01153.x

[22] Wu, Y. et al. Reliability and accuracy of dynamic navigation for zygomatic implant placement. *Clin. Oral. Implants Res.* **33**, 362–376 (2022). 35113463 10.1111/clr.13897 PMC9305866

[23] Malhotra, S. et al. Augmented reality in surgical navigation: a review of evaluation and validation metrics. *Appl. Sci.* **13**, 1629 (2023).

[24] Vajsbaher, T., Schultheis, H. & Francis, N. K. Spatial cognition in minimally invasive surgery: a systematic review. *BMC Surg.* **18**, 94 (2018). 30404634 10.1186/s12893-018-0416-1 PMC6223063

[25] Saylany, A. et al. The use of a novel heads-up display (HUD) to view intra-operative x-rays during a one-level cervical arthroplasty. *World Neurosurg.* **138**, 369–373 (2020). 32201294 10.1016/j.wneu.2020.03.073

[26] Burström, G., Persson, O., Edström, E. & Elmi-Terander, A. Augmented reality navigation in spine surgery: a systematic review. *Acta Neurochir.* **163**, 843–852 (2021). 33506289 10.1007/s00701-021-04708-3 PMC7886712

[27] Müller, F. et al. Augmented reality navigation for spinal pedicle screw instrumentation using intraoperative 3D imaging. *Spine J. Off. J. North Am. Spine Soc.* **20**, 621–628 (2020). 10.1016/j.spinee.2019.10.012 31669611

[28] Frisk, H. et al. Feasibility and accuracy of thoracolumbar pedicle screw placement using an augmented reality head mounted device. *Sensors (Basel, Switzerland)* **22**10.3390/s22020522 (2022). 10.3390/s22020522 PMC8779462 35062483

[29] Condino, S. et al. How to Build a Patient-Specific Hybrid Simulator for Orthopaedic Open Surgery: Benefits and Limits of Mixed-Reality Using the Microsoft HoloLens. *J. Healthc. Eng.* **2018**, 5435097 (2018). 30515284 10.1155/2018/5435097 PMC6236521

[30] Wolf, J. et al. How different augmented reality visualizations for drilling affect trajectory deviation, visual attention, and user experience. *Int. J. Comput. Assist. Radiol. Surg.*10.1007/s11548-022-02819-5 (2023). 10.1007/s11548-022-02819-5 PMC10363038 36808552

[31] Tu, P. et al. Augmented reality based navigation for distal interlocking of intramedullary nails utilizing Microsoft HoloLens 2. *Comput. Biol. Med.* **133**, 104402 (2021). 33895460 10.1016/j.compbiomed.2021.104402

[32] Tu, P., Wang, H., Joskowicz, L. & Chen, X. A multi-view interactive virtual-physical registration method for mixed reality based surgical navigation in pelvic and acetabular fracture fixation. *Int. J. Comput. Assist. Radiol. Surg.*10.1007/s11548-023-02884-4 (2023). 10.1007/s11548-023-02884-4 37031310

[33] Kriechling, P. et al. Augmented reality through head-mounted display for navigation of baseplate component placement in reverse total shoulder arthroplasty: a cadaveric study. *Arch. Orthop. trauma Surg.* **143**, 169–175 (2023). 34213578 10.1007/s00402-021-04025-5 PMC9886637

[34] Mai, H.-N., van Dam, V. & Lee, D.-H. Accuracy of augmented reality-assisted navigation in dental implant surgery: systematic review and meta-analysis. *J. Med. Internet Res.* **25**, e42040 (2023). 36598798 10.2196/42040 PMC9856431

[35] Móga, K., Ferencz, A. & Haidegger, T. What is next in computer-assisted spine surgery? Advances in image-guided robotics and extended reality. *Robotics* **12**, 1 (2023).

[36] Móga, K., Hölgyesi, Á., Zrubka, Z., Péntek, M. & Haidegger, T. Augmented or mixed reality enhanced head-mounted display navigation for in vivo spine surgery: a systematic review of clinical outcomes. *J. Clin. Med.* **12**10.3390/jcm12113788 (2023). 10.3390/jcm12113788 PMC10254054 37297990

[37] Pellegrino, G. et al. Augmented reality for dental implantology: a pilot clinical report of two cases. *BMC Oral. Health* **19**, 158 (2019). 31324246 10.1186/s12903-019-0853-y PMC6642526

[38] Gertzbein, S. D. & Robbins, S. E. Accuracy of pedicular screw placement in vivo. *Spine* **15**, 11–14 (1990). 2326693 10.1097/00007632-199001000-00004

[39] Liebmann, F. et al. Pedicle screw navigation using surface digitization on the Microsoft HoloLens. *Int. J. Comput. Assist. Radiol. Surg.* **14**, 1157–1165 (2019). 30993519 10.1007/s11548-019-01973-7

[40] Kleck, C. J. et al. A new 3-dimensional method for measuring precision in surgical navigation and methods to optimize navigation accuracy. *Eur. Spine J.* **25**, 1764–1774 (2016). 26394858 10.1007/s00586-015-4235-0

[41] Tao, B. et al. Comparative accuracy of cone-beam CT and conventional multislice computed tomography for real-time navigation in zygomatic implant surgery. *Clin. Implant Dent. Relat. Res.* **22**, 747–755 (2020). 33112508 10.1111/cid.12958

[42] Paraskevopoulos, D. et al. Comparative study of application accuracy of two frameless neuronavigation systems: experimental error assessment quantifying registration methods and clinically influencing factors. *Neurosurg. Rev.* **34**, 217–228 (2010). 21246391 10.1007/s10143-010-0302-5

[43] Lavallee, S. et al. Method of auto-calibration and auto-registration of an intra-operative 3D imaging system integrated with navigation. *CAOS 2018* **2**, 127–129 (2018).

[44] Hecht, N. et al. Accuracy and workflow of navigated spinal instrumentation with the mobile AIRO(®) CT scanner. *Eur. Spine J.* **25**, 716–723 (2016). 25702317 10.1007/s00586-015-3814-4

[45] Frisk, H. et al. Automatic image registration on intraoperative CBCT compared to Surface Matching registration on preoperative CT for spinal navigation: accuracy and workflow. *Int. J. Comput. Assist. Radiol. Surg.* **19**, 665–675 (2024). 38378987 10.1007/s11548-024-03076-4 PMC10973038

[46] Costa, G. M., Petry, M. R., Martins, J. G. & Moreira, A. P. G. M. Assessment of multiple fiducial marker trackers on hololens 2. *IEEE Access* **12**, 14211–14226 (2024).

[47] Sorriento, A. et al. Optical and electromagnetic tracking systems for biomedical applications: a critical review on potentialities and limitations. *IEEE Rev. Biomed. Eng.* **13**, 212–232 (2020). 31484133 10.1109/RBME.2019.2939091

[48] Condino, S., Carbone, M., Piazza, R., Ferrari, M. & Ferrari, V. Perceptual limits of optical see-through visors for augmented reality guidance of manual tasks. *IEEE Trans. Bio Med. Eng.* **67**, 411–419 (2020). 10.1109/TBME.2019.2914517 31059421

[49] Chegini, S., Edwards, E., McGurk, M., Clarkson, M. & Schilling, C. Systematic review of techniques used to validate the registration of augmented-reality images using a head-mounted device to navigate surgery. *Br. J. Oral. Maxillofac. Surg.* **61**, 19–27 (2023). 36513525 10.1016/j.bjoms.2022.08.007

[50] Hu, X., Baena, F. R. Y. & Cutolo, F. Head-mounted augmented reality platform for markerless orthopaedic navigation. *IEEE J. Biomed. Health Inform.* **26**, 910–921 (2022). 34115600 10.1109/JBHI.2021.3088442

[51] Doughty, M., Ghugre, N. R. & Wright, G. A. Augmenting performance: a systematic review of optical see-through head-mounted displays in surgery. *J. Imag.* **8**10.3390/jimaging8070203 (2022). 10.3390/jimaging8070203 PMC9318659 35877647

[52] Bertollo, N. & Robert, W. Drilling of bone: practicality, limitations and complications associated with surgical drill-bits. In *Biomechanics in Applications*, edited by V. Klika (InTech2011).

[53] Bertollo, N., Gothelf, T. K. & Walsh, W. R. 3-Fluted orthopaedic drills exhibit superior bending stiffness to their 2-fluted rivals: clinical implications for targeting ability and the incidence of drill-bit failure. *Injury* **39**, 734–741 (2008). 18490018 10.1016/j.injury.2007.11.286

[54] Koop, R., Vercruyssen, M., Vermeulen, K. & Quirynen, M. Tolerance within the sleeve inserts of different surgical guides for guided implant surgery. *Clin. Oral. Implants Res.* **24**, 630–634 (2013). 22413853 10.1111/j.1600-0501.2012.02436.x

[55] Augustin, G. et al. Cortical bone drilling and thermal osteonecrosis. *Clin. Biomech. (Bristol, Avon)* **27**, 313–325 (2012). 22071428 10.1016/j.clinbiomech.2011.10.010

[56] Qin, D., Zhang, Q., Zhang, Y.-Z., Pan, J.-S. & Chen, W. Safe drilling angles and depths for plate-screw fixation of the clavicle: avoidance of inadvertent iatrogenic subclavian neurovascular bundle injury. *J. Trauma* **69**, 162–168 (2010). 20068484 10.1097/TA.0b013e3181bbd617

[57] Scherer, U., Stoetzer, M., Ruecker, M., Gellrich, N.-C. & von See, C. Template-guided vs. non-guided drilling in site preparation of dental implants. *Clin. Oral. Investig.* **19**, 1339–1346 (2015). 25354488 10.1007/s00784-014-1346-7

[58] Zhan, T. et al. Practical chromatic aberration correction in virtual reality displays enabled by cost‐effective ultra‐broadband liquid crystal polymer lenses. *Adv. Opt. Mater.* **8**10.1002/adom.201901360 (2020).

[59] Johari, K., Ouden, D.-Bden & Behroozmand, R. Effects of aging on temporal predictive mechanisms of speech and hand motor reaction time. *Aging Clin. Exp. Res.* **30**, 1195–1202 (2018). 29392576 10.1007/s40520-018-0902-4 PMC6070444

[60] Schulz, K. F., Altman, D. G. & Moher, D. CONSORT 2010 statement: updated guidelines for reporting parallel group randomised trials. *BMJ (Clin. Res. ed.)* **340**, c332 (2010). 10.1136/bmj.c332 PMC2844940 20332509

[61] Dwan, K., Li, T., Altman, D. G. & Elbourne, D. CONSORT 2010 statement: extension to randomised crossover trials. *BMJ (Clin. Res. ed.)* **366**, l4378 (2019). 10.1136/bmj.l4378 PMC6667942 31366597

[62] Ng, A. W. Y. & Chan, A. H. S. Color associations among designers and non-designers for common warning and operation concepts. *Appl. Ergon.* **70**, 18–25 (2018). 29866309 10.1016/j.apergo.2018.02.004

[63] Boudissa, M., Prod’homme, M., Kerschbaumer, G., Ruatti, S. & Tonetti, J. 3D-imaging in percutaneous spine surgery using the Surgivisio system. *Orthop. Traumatol. Surg. Res.* **106**, 1183–1186 (2020). 32893168 10.1016/j.otsr.2020.01.018

[64] Kuznetsova, A., Brockhoff, P. B. & Christensen, R. H. B. lmerTest Package: tests in linear mixed effects models. *J. Stat. Soft*. **82**10.18637/jss.v082.i13 (2017).

