# Supplementary material for: Accuracy and efficiency of drilling trajectories with augmented reality versus conventional navigation randomized crossover trial
Source: NPJ Digit Med. 2024 Nov 10;7:316. doi: 10.1038/s41746-024-01314-2 (PMC11551163; doi:10.1038/s41746-024-01314-2)
Supplement: Supplementary file 1 — Supplementary Information [file 41746_2024_1314_MOESM1_ESM.pdf]

Supplementary Information

Figures

Supplementary Figure 1 – Visualization of drilling accuracy by method

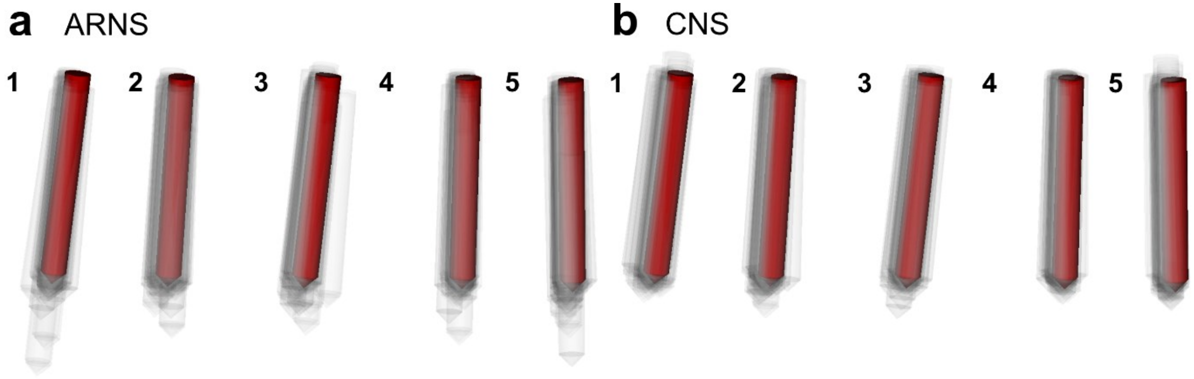

Supplementary Figure 1. (a-b) Comparison of conducted trajectories 1 to 5 in red of Augmented Reality Navigation System (ARNS) (**a**) and Conventional Navigation System (CNS) (**b**) with planning trajectories (grey transparent).

Movies

Supplementary Movie 1 – Demonstration of both systems

This movie begins with an introduction to the general experimental setup, followed by a presentation of the setup and guidance function of the Augmented Reality Navigation System (ARNS). This is followed by a demonstration of ARNS guidance during the drilling process, showing a virtual twin guiding the drilling. The video then switches to the Conventional Navigation System (CNS), showing first the setup and then the guidance function of the CNS. It then shows the CNS screen during guided drilling.

Tables

Supplementary Table 1 – Sub-analysis of surgeons

		Sub-analysis of the surgeons								
		Previous Experience in CNS		Surgical Experience (Median)						
		Yes (n = 90/9)	No (n = 30/3)	Higher (n = 60/6)	Lower (n = 60/6)	Surgeons (n = 120/12)	Students (n = 120/12)	Engineers (n = 120/12)	Total (n = 360/36)	p value*
Maximum Projected Translational Deviation (mm)	Mean (SD)	**0.96 (0.49)**	0.97 (0.34)	0.98 (0.40)	**0.96 (0.45)**	**0.96 (0.45)**	1.05 (0.46)	1.21 (0.48)	1.07 (0.48)	0.319 / 0.004
Projected Translational Deviation at the Entry Points (mm)	Mean (SD)	**0.82 (0.39)**	0.85 (0.33)	0.86 (0.32)	**0.82 (0.38)**	**0.82 (0.38)**	0.93 (0.44)	1.09 (0.44)	0.95 (0.43)	0.203 / 0.002
Projected Translational Deviation at the Endpoints (mm)	Mean (SD)	**0.87 (0.53)**	0.89 (0.39)	0.88 (0.45)	**0.87 (0.50)**	**0.87 (0.50)**	0.93 (0.51)	1.07 (0.52)	0.96 (0.51)	0.513 / 0.039
Translational Deviation in 3D Euclidean Distance at Entry Points (mm)	Mean (SD)	**0.83 (0.40)**	0.86 (0.34)	0.87 (0.33)	**0.84 (0.38)**	**0.84 (0.38)**	0.94 (0.45)	1.11 (0.44)	0.96 (0.44)	0.205 / 0.002
Angular Deviation (°)	Mean (SD)	0.88 (0.57)	0.81 (0.52)	**0.79 (0.43)**	0.87 (0.55)	**0.87 (0.55)**	0.95 (0.60)	0.94 (0.44)	0.92 (0.54)	0.435 / 0.523
Depth Deviation (mm)	Mean (SD)	1.29 (1.51)	**0.97 (1.00)**	0.99 (0.77)	1.21 (1.40)	1.21 (1.40)	**0.59 (0.87)**	0.90 (1.52)	0.90 (1.32)	0.024 / 0.0256
NASA-TLX	ARNS	47.1 (20.5)	**43.2 (8.8)**	56.3 (16.4)	**36.0 (14.0)**	**46.1 (18.0)**	55.7 (16.6)	47.2 (11.5)	49.7 (15.8)	0.275
	CNS	57.1 (16.6)	**34.8 (2.4)**	52.0 (13.4)	**51.0 (22.1)**	51.5 (17.4)	63.8 (11.2)	**50.9 (12.6)**	55.4 (14.9)	0.051
SUS	ARNS	**81.1 (12.4)**	61.7 (16.1)	71.2 (13.1)	**81.2 (16.9)**	76.2 (15.4)	**73.1 (15.7)**	83.5 (15.7)	77.6 (15.8)	0.259
	CNS	65.8 (23.3)	**79.2 (10.4)**	**74.2 (12.5)**	64.2 (27.8)	69.2 (21.2)	**56.0 (19.1)**	68.8 (15.2)	64.7 (19.2)	0.164
Previous Experience with CNS	no	7 (77.8%)	2 (66.7%)	2 (33.3%)	1 (16.7%)	3 (25.0%)	12 (100.0%)	9 (75.0%)	24 (66.7%)	< 0.001
	yes	2 (22.2%)	1 (33.3%)	4 (66.7%)	5 (83.3%)	9 (75.0%)	0 (0.0%)	3 (25.0%)	12 (33.3%)	
Previous Experience with AR	no	0 (0%)	3 (100%)	4 (66.7%)	5 (83.3%)	9 (75.0%)	10 (83.3%)	6 (50.0%)	25 (69.4%)	0.182
	yes	9 (100%)	0 (0%)	2 (33.3%)	1 (16.7%)	3 (25.0%)	2 (16.7%)	6 (50.0%)	11 (30.6%)	
Preferred Navigation System	ARNS	7 (77.8%)	0 (0.0%)	3 (50.0%)	4 (66.7%)	7 (58.3%)	10 (83.3%)	10 (83.3%)	27 (75.0%)	0.264
	CNS	2 (22.2%)	3 (100.0%)	3 (50.0%)	2 (33.3%)	5 (41.7%)	2 (16.7%)	2 (16.7%)	9 (25.0%)	

n = number of trajectories drilled / number of participants; * = if two p-values, first for students, second for engineers, reference surgeons.

Supplementary Table 2 – Likert Questionnaire

#	Likert questions	ARNS				CNS					
		Surgeon (n=12)	Student (n=12)	Engineer (n=12)	ARNS Total (n=36)	Surgeon (n=12)	Student (n=12)	Engineer (n=12)	CNS Total (n=36)	Total (n=72)	p*
1.	The (AR-based | conventional) navigation system interfered with my drilling trajectory.	1.5 (0.8)	1.6 (0.7)	1.4 (0.7)	1.5 (0.7)	1.9 (1.2)	2.1 (0.7)	1.3 (0.7)	1.8 (0.9)	1.6 (0.8)	0.065
2.	With the (AR-based | conventional) navigation system, it is easy to find the starting point.	3.2 (0.8)	3.4 (0.7)	3.5 (0.7)	3.4 (0.7)	2.8 (1.3)	2.5 (0.8)	3.0 (1.0)	2.8 (1.0)	3.1 (0.9)	0.473
3.	With the (AR-based | conventional) navigation system, it is easy to set the orientation.	3.3 (0.8)	3.5 (0.7)	3.4 (0.9)	3.4 (0.8)	2.8 (0.9)	2.8 (0.9)	3.2 (0.6)	2.9 (0.8)	3.2 (0.8)	0.57
4.	The (AR-based | conventional) navigation system is easy to use.	3.4 (1.0)	3.3 (0.7)	3.6 (0.7)	3.4 (0.8)	3.0 (1.2)	2.7 (1.0)	3.2 (0.6)	2.9 (1.0)	3.2 (0.9)	0.316
5.	I felt safe using the (AR-based | conventional) navigation system.	3.2 (0.7)	2.9 (0.5)	3.5 (0.7)	3.2 (0.7)	3.2 (0.9)	2.4 (0.8)	2.9 (0.9)	2.8 (0.9)	3.0 (0.8)	**0.04**
6.	The (AR-based | conventional) navigation system is good for drilling trajectories.	3.6 (0.7)	3.6 (0.7)	3.2 (0.8)	3.5 (0.7)	2.9 (1.1)	2.8 (0.4)	3.1 (0.5)	2.9 (0.7)	3.2 (0.7)	0.623
7.	I find the (AR-based | conventional) navigation system unnecessarily complex.	1.8 (0.8)	1.4 (0.7)	1.7 (0.8)	1.6 (0.8)	2.2 (1.2)	2.1 (0.8)	1.5 (0.5)	1.9 (0.9)	1.8 (0.8)	0.398
8.	I think I would need the assistance of a technical person to use the (AR-based | conventional) navigation system.	2.0 (1.0)	2.5 (0.9)	1.8 (0.6)	2.1 (0.9)	1.8 (1.1)	2.6 (1.0)	1.9 (0.8)	2.1 (1.0)	2.1 (0.9)	**0.015**
9.	I think that the various functions in the (AR-based | conventional) navigation system were well integrated.	3.2 (1.0)	3.3 (0.7)	3.5 (0.8)	3.4 (0.8)	2.8 (0.8)	2.8 (0.5)	2.8 (1.0)	2.8 (0.8)	3.1 (0.8)	0.629
10.	I still have a lot to learn before I could use the (AR-based | conventional) navigation system.	2.0 (0.9)	2.4 (0.7)	1.6 (0.7)	2.0 (0.8)	2.2 (1.0)	2.9 (0.9)	1.9 (1.1)	2.4 (1.0)	2.2 (0.9)	**0.002**
11.	I found the (AR-based | conventional) navigation system intuitive.	3.2 (1.0)	3.5 (0.7)	3.6 (0.5)	3.4 (0.7)	2.9 (1.1)	2.7 (0.7)	2.8 (0.8)	2.8 (0.9)	3.1 (0.9)	0.818
12.	I drilled the trajectories accurately with the (AR-based | conventional) navigation system.	2.8 (0.6)	2.8 (0.5)	3.1 (0.9)	2.9 (0.7)	2.9 (1.0)	2.2 (0.6)	2.5 (0.8)	2.5 (0.8)	2.7 (0.8)	0.118

* Kruskal-Wallis test comparing total responses of surgeons, students, and engineers. Only questions 5, 8 and 10 showed significant differences between the professions.
